# The extracellular matrix protein laminin-10 promotes blood–brain barrier repair after hypoxia and inflammation in vitro

**DOI:** 10.1186/s12974-016-0495-9

**Published:** 2016-02-01

**Authors:** Korakoch Kangwantas, Emmanuel Pinteaux, Jeffrey Penny

**Affiliations:** Manchester Pharmacy School, University of Manchester, Manchester, M13 9PT UK; Faculty of Life Sciences, University of Manchester, A.V. Hill Building, Oxford Road, Manchester, M13 9PT UK

**Keywords:** Blood–brain barrier, Laminin, Extracellular matrix, Cerebrovasculature, Endothelia, Inflammation, Interleukin, Hypoxia

## Abstract

**Background:**

The blood–brain barrier (BBB) of the central nervous system (CNS) is essential for normal brain function. However, the loss of BBB integrity that occurs after ischaemic injury is associated with extracellular matrix (ECM) remodelling and inflammation, and contributes to poor outcome. ECM remodelling also contributes to BBB repair after injury, but the precise mechanisms and contribution of specific ECM molecules involved are unknown. Here, we investigated the mechanisms by which hypoxia and inflammation trigger loss of BBB integrity and tested the hypothesis ECM changes could contribute to BBB repair in vitro.

**Methods:**

We used an in vitro model of the BBB, composed of primary rat brain endothelial cells grown on collagen (Col) I-, Col IV-, fibronectin (FN)-, laminin (LM) 8-, or LM10-coated tissue culture plates, either as a single monolayer culture or on Transwell® inserts above mixed glial cell cultures. Cultures were exposed to oxygen-glucose deprivation (OGD) and/or reoxygenation, in the absence or the presence of recombinant interleukin-1β (IL-1β). Cell adhesion to ECM molecules was assessed by cell attachment and cell spreading assays. BBB dysfunction was assessed by immunocytochemistry for tight junction proteins occludin and zona occludens-1 (ZO-1) and measurement of trans-endothelial electrical resistance (TEER). Change in endothelial expression of ECM molecules was assessed by semi-quantitative RT-PCR.

**Results:**

OGD and/or IL-1 induce dramatic changes associated with loss of BBB integrity, including cytoplasmic relocalisation of membrane-associated tight junction proteins occludin and ZO-1, cell swelling, and decreased TEER. OGD and IL-1 also induced gene expression of key ECM molecules associated with the BBB, including FN, Col IV, LM 8, and LM10. Importantly, we found that LM10, but not FN, Col IV, nor LM8, plays a key role in maintenance of BBB integrity and reversed most of the key hallmarks of BBB dysfunction induced by IL-1.

**Conclusions:**

Our data unravel new mechanisms of BBB dysfunction induced by hypoxia and inflammation and identify LM10 as a key ECM molecule involved in BBB repair after hypoxic injury and inflammation.

## Background

The blood–brain barrier (BBB) of the central nervous system (CNS) is a specialised vascular structure that is essential for normal brain function and homeostasis (see [1] for review). It is composed of brain endothelial cells (BEC), pericytes, and astrocyte endfeet that are embedded in a network of extracellular matrix (ECM) molecules that form the basement membrane of the cerebrovasculature (see [2, 3] for review). In a physiological state, BBB integrity is essential to protect brain tissue from blood-derived toxins and to limit circulating immune cell infiltration whilst allowing selective transport of nutrients that are essential for neuronal activity. BBB integrity is primarily maintained by close interaction of BEC triggered by tight junctions that restrict paracellular transport. Tight junctions are composed of cell surface proteins including occludins and claudins that interact in a homodimeric fashion, as well as intracellular adaptor proteins such as zona occludens-1 (ZO-1). Integrity of the BBB is also maintained by attachment of BEC to blood vessel basement membrane, the latter being composed of large glycoprotein ECM molecules such as fibronectin (FN), collagen (Col) IV, and laminin (LM) isoforms, of which LM 8 (α4β1γ1) and LM 10 (α5β1γ1) are primarily found in the basement membrane of the cerebrovasculature [4, 5]. BEC interaction with the basement membrane occurs via integrins, the major cell surface adhesion molecules responsible for anchoring endothelial cells to the ECM [3, 6] that are also critically involved in the interaction between the basement membrane and astrocyte endfeet, thereby further contributing to BBB integrity [3].

In contrast, acute CNS disorders, such as cerebral ischaemia, result in loss of BBB integrity, which is known to contribute to brain injury and is associated with poor outcome [7]. Key events associated with BBB dysfunction include rapid degradation of ECM molecules, loss of interaction between endothelial cells and the basement membrane, and disruption of tight junctions, leading to a leaky BBB that allows extravasation of systemic circulating blood-derived molecules and leucocytes [8]. These events are also associated with cerebrovascular inflammation primarily driven by the pro-inflammatory cytokine interleukin-1 (IL-1). IL-1 induces endothelial cell activation [9] and neutrophil infiltration [10], which contributes to neuronal injury. However the precise events that cause BBB dysfunction are unclear, and it is unknown whether IL-1 directly contributes to these events. Degradation of the ECM network following injury is followed by synthesis and deposition of de novo expressed ECM molecules, which is associated with ECM remodelling and BBB repair mechanisms post injury, although the mechanisms involved and the specific contribution of ECM to BBB repair are completely unknown. Using an in vitro BBB model, we demonstrate that oxygen-glucose deprivation (OGD) and IL-1 are key triggers for BBB dysfunction as well as de novo expression of ECM molecules and that LM10 is a key ECM molecule involved in BBB repair.

## Methods

### Extracellular matrix proteins and plate coating

Mouse LM (isoform -8 (α4β1γ1) and -10 (α5β1γ1)) (Biolamina, UK) and human plasma FN (Chemicon, UK) were diluted in PBS containing Ca^2+^ and Mg^2+^ (PBS^+^) (Invitrogen, UK), and rat tail Col I and Col IV (BD Biosciences, UK) were diluted in 1 % (v/v) acetic acid to the required concentrations recommended by the manufacturer’s instructions. Tissue culture plates (Corning, UK) were pre-coated with 0.1 % (w/v) bovine serum albumin (BSA) in PBS^+^ (control wells) or with ECM molecules (100 nM) for 1 h at room temperature, or at 4 °C overnight. Non-specific cell-binding sites were then blocked with filter sterilised (0.22 μm pore size filter, Nalgene, UK) 0.1 % (w/v) BSA in PBS without Ca^2+^ or Mg^2+^ (PBS^−^) at room temperature for 1 h, and plates were washed in PBS^−^ three times before cell seeding.

### Cell cultures

Primary rat brain endothelial cell (rBEC) cultures were prepared from 6–8 Sprague-Dawley adult rat brains, as described previously [8]. Cells were grown in tissue culture flasks (T25 Corning, UK) in Dulbecco’s modified Eagle’s medium (DMEM, Invitrogen, UK) supplemented with 20 % (v/v) plasma-derived serum (PDS, First Link Ltd, UK), 2 mM glutamine, 80 μg/ml heparin, 0.5 μg/ml basic fibroblast growth factor, 1 % (v/v) rBEC supplement (to a final concentration of 5 μg/ml ascorbic acid, 5 μg/ml insulin, 5 μg/ml transferrin, 5 ng/ml sodium selenite and 325 μg/ml glutathione), 100 units/ml penicillin and 0.1 μg/ml streptomycin, in a 5 % CO_2_ humidified incubator at 37 °C until cultures reached confluency (10–12 days in vitro, DIV). The purity of the cultures was at least 99 %, as assessed by Von Willebrand factor immunostaining (data not shown).

Primary rat astrocyte cultures were prepared from 1–3-day-old Sprague-Dawley rat pups as described previously [11, 12]. Cells were plated in the lower chamber of Transwell® inserts pre-coated with poly-D-lysine (Sigma, UK) at a density of 10,000 cells/cm^2^ and were grown in DMEM supplemented with 10 % (v/v) heat inactivated foetal bovine serum (FBS, Lonza Sales AG), 100 units/ml penicillin, and 0.1 μg/ml streptomycin, in a 5 % CO_2_ humidified incubator at 37 °C. Cells were grown for 3 to 4 days before being co-cultured with rBEC cultures. The purity of the cultures was 98 %, as assessed by glial fibrillary acidic protein (GFAP) immunostaining (data not shown).

### Generation of the rat in vitro BBB model

Medium from primary rat astrocytes grown in the lower chamber of Transwell® plates were completely removed and replaced with rBEC growth medium. Transwell® inserts (0.4 μm pore size polyester membrane) were then inserted into the appropriate wells and rBECs; obtained by trypsinisation of confluent rBEC, cultures grown in T25 flasks were seeded at a density of 75,000 cells/cm^2^ onto ECM-coated Transwell® inserts. The rat BBB model was maintained in a 5 % CO_2_ humidified incubator at 37 °C. The medium was changed 24 h post seeding and every 2 days until confluency (4–5 DIV).

### Cell adhesion assays

Cell adhesion to the ECM was assessed using cell attachment and cell spreading assays as previously reported [8].

### Oxygen-glucose deprivation and interleukin-1β treatment

In order to mimic the ischaemic and inflammatory conditions that occur during stroke, the in vitro BBB model was exposed to oxygen-glucose deprivation (OGD) with or without reoxygenation, and in the absence or the presence of IL-1β, as follows. To produce OGD conditions, rBEC-astrocyte co-cultures were placed in an airtight hypoxic glove box. Nitrogen gas was used to purge other gases from the hypoxic glove box, which was then placed in an airtight chamber at 37 °C with CO_2_ and O_2_ levels set at 5 and 1 %, respectively. The original glucose-containing rBEC growth medium was removed, and wells were washed once with glucose-free DMEM before addition of glucose-free DMEM medium to each well. Cultures were exposed to OGD for 2.5 h. The OGD condition was terminated by removing the cultures from the hypoxic chamber and changing the culture medium to glucose-containing medium (1 g/l) supplemented with PDS (20 % v/v) for different times of reoxygenation. Control cultures were maintained in normoxic conditions in DMEM-containing glucose for 2.5 h. For induction of inflammation, recombinant rat IL-1β (R&D Systems, UK), dissolved in vehicle composed of 0.1 % (w/v) low endotoxin BSA in 0.9 % (w/v) sodium chloride, was added to the upper Transwell® compartment for different amounts of time at a final concentration of 10 ng/ml, and at the start of reoxygenation for a period of 2 h for the OGD experiments.

### Immunocytochemistry

The polyester membranes of Transwell® inserts were cut out of the insert cases with a scalpel. Cells were then rinsed three times with PBS, permeabilised with 0.1 % (v/v) Triton® X-100 and 1.5 mM sodium azide in PBS for 10 min, rinsed with PBS, and incubated with 0.25 % (w/v) NH_4_Cl in PBS for 5 min. Cells were incubated with 1 % (v/v) donkey serum for 1 h in order to block all non-specific binding sites and were subsequently incubated with primary antibodies; rabbit anti-human ZO-1, or mouse anti-human occludin antibodies (Invitrogen, UK) at a final concentration of 0.25 μg/ml. Cells were then incubated for 1 h with Alexa Fluor® 594-conjugated donkey anti-mouse IgG antibody (Invitrogen, UK) or Alexa Fluor® 488-conjugated donkey anti-rabbit IgG antibody (Invitrogen, UK) at a working dilution of 1:1000. Images were acquired on a Delta Vision RT (Applied Precision, UK) restoration microscope using a *60× 1.42 Plan Apo* objective or using an Olympus BX51 upright microscope equipped with 4×/0.13 Plan fln, 10×/0.3 Plan fln and 20×/0.5 Plan fln objectives. The images were collected using a Coolsnap HQ camera (Photometrics, UK) with a Z optical spacing of 0.2 μm and processed using ImageJ software 1.37v (National Institutes of Health, USA). The mean intensity and the ratio of the mean cytoplasmic intensity to the mean membranous intensity were quantified optically using ImageJ. Circularity of cells was calculated automatically by the ImageJ software using the formula

$$ \mathrm{Circularity} = 4 \times \left(\mathrm{area}/{\mathrm{perimeter}}^2\right). $$

A circularity value of 1.0 indicated a perfect circle whereas a value of 0.0 indicated an elongated shape.

### Measurement of trans-endothelial electrical resistance (TEER)

An EVOM™ Epithelial Voltohmmeter and chopstick electrodes (World Precision Instruments, UK) were used for the measurement of the electrical resistance of brain endothelial cell monolayers grown on Transwell® inserts. Monolayer TEER was calculated by subtracting the value of a blank Transwell® insert from the total resistance obtained for the endothelial monolayer and insert, and each TEER value was normalised for the surface area of the insert.

### Reverse-transcriptase polymerase chain reaction (RT-PCR)

Total RNA was isolated from rBECs using the ISOLATE RNA mini kit (Bioline, UK) according to the manufacturer’s instruction, and the concentration and purity of RNAs were determined by measuring the absorbance at 260 nm and the 260/280 nm ratio, respectively, by Nanodrop (Thermo Scientific, UK). mRNAs were reverse transcribed to cDNA using the cDNA synthesis kit (Bioline, UK). cDNAs (1 μl) were then amplified in a GeneAmp® PCR system 9700 in the presence of 5 μl Mytaq red mix (Bioline, UK), 200 nM forward primer, 200 nM reverse primer (Eurofins MWG operon, primer sequences are found in Table 1), and RNase-free water (to a total volume of 50 μl). PCR reactions were then conducted for *lmα4*, *lmα5*, *lmβ1*, *lmγ1*,*col4*, *fn*, and the housekeeping gene *Rpl13a* (ribosomal protein L13A) as follows: 1 min at 95 °C [15 s at 95 °C, 15 s at annealing temperature (53 °C), 10 s at 72 °C] for 18–38 cycles, and a 5-min final extension period at 72 °C. The cycle number for each reaction was chosen to be in the linear phase of amplification. PCR products were separated by electrophoresis using an ethidium bromide-containing 1.5 % (w/v) agarose gel and were quantified using GE healthcare ImageQuant™. Control PCR reactions for each pair of primers used total RNA to ensure that PCR products were not due to genomic DNA amplification.

**Table 1:** Primer sequences for RT-PCR

Gene	Primer	Sequence (5′ to 3′)	Product size (bp)
Lmα4	Forward	AGAATGTTCGCCCTGTGACT	437
	Reverse	CCAGGAGCACATCTTTCACA	
Lmα5	Forward	GACTCGCCTCATGTCTGTCA	424
	Reverse	TGCACTGGCCTGACTCTG	
Lmβ1	Forward	TCCTGACAGCCATCTCATTG	371
	Reverse	AAGCAGGATCTAAGGCACGA	
Lmγ1	Forward	GCAGCCTTCTTGACCGACTA	379
	Reverse	GCCTTCCTTCCAGAGTTGAA	
FN	Forward	CACATGGAGCAAGAAGGACA	257
	Reverse	AGGTGAACGGGAGAACACAG	
Col IV	Forward	AAAGGAGAGCGAGGCTACC	349
	Reverse	GTCCAACTTCGCCTGTCAA	
Rpl13A	Forward	GAGGGCATCAACATTTCTGG	428
	Reverse	GCCTGTTTCCTTAGCCTCAA	

### Statistical analysis

Data were analysed using GraphPad Prism. Data were analysed by one sample repeated *t* test, one-way analysis of variance (ANOVA) with the Tukey post hoc test or by two-way ANOVA with the Bonferroni post hoc test. The level of significance was represented as *p < 0.05, **p < 0.01, and ***p < 0.001.

## Results

### OGD and IL-1 induce loss of BBB integrity in vitro

**Fig. 1:**
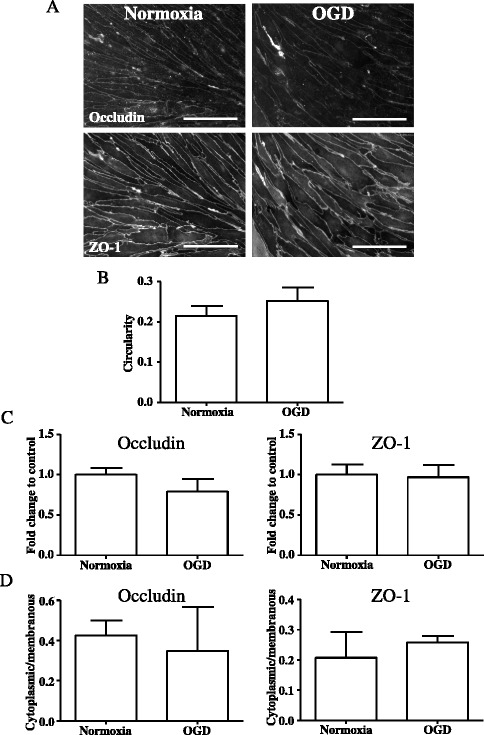
Primary rBEC cultures on Col I-coated Transwell® inserts were subjected to normoxia or OGD for 2.5 h. Immunocytochemical images for occludin and ZO-1 (**a**) were analysed with ImageJ to measure cell circularity (**b**), total intensity of fluorescence (**c**), and ratio of mean cytoplasmic fluorescence intensity to mean membranous ZO-1 fluorescence intensity (**d**). Data are shown as mean ± standard deviation from three independent experiments carried out on separate cultures. Data were statistically analysed by one-way ANOVA and Bonferroni’s post hoc tests to compare all data sets. The *scale bar* in **a** represents 100 μm

**Fig. 2:**
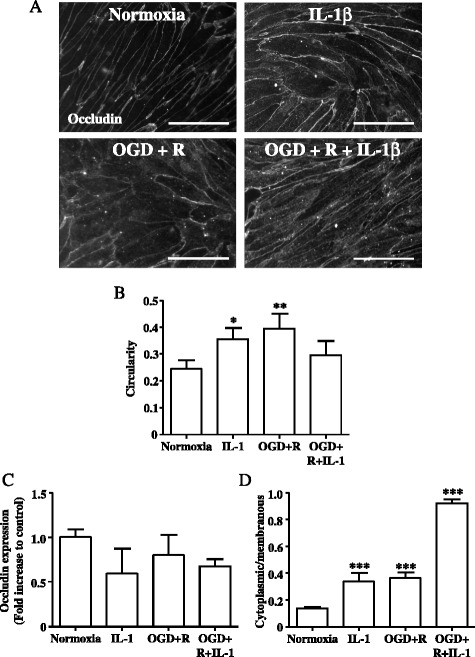
Primary rBEC cultures on Col I-coated Transwell® inserts were subjected to normoxia, IL-1β treatment for 4 h, 2.5 h OGD and 4 h reoxygenation (OGD + R), 2.5 h OGD, and 4 h reoxygenation in the presence of IL-1β (OGD + R + IL-1). Immunocytochemical images for occludin (**a**) were analysed with ImageJ to measure cell circularity (**b**), total intensity of occludin fluorescence (**c**), and ratio of mean cytoplasmic fluorescence intensity to mean membranous occludin fluorescence intensity (**d**). Data are shown as mean ± standard deviation from three independent experiments carried out on separate cultures. Data were statistically analysed by one-way ANOVA and Bonferroni’s post hoc tests to compare all data sets. *p < 0.05, **p < 0.01, and ***p < 0.001 vs. normoxia. *The scale* bar in **a** represents 100 μm

**Fig. 3:**
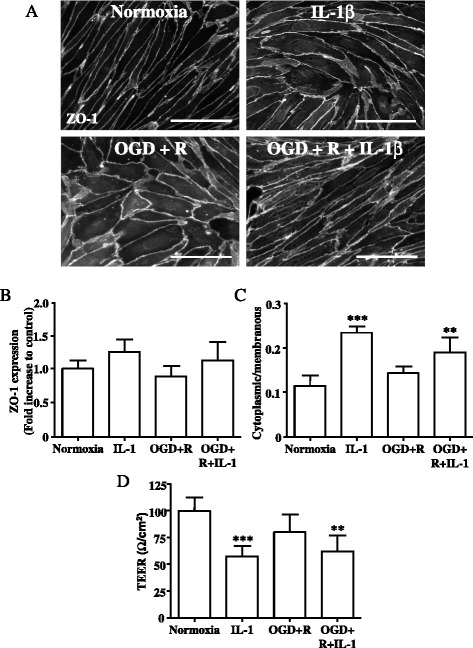
Primary rBEC cultures on Col I-coated Transwell® inserts were subjected to normoxia, IL-1β treatment for 4 h (IL-1), 2.5 h OGD and 4 h reperfusion (OGD + R), 2.5 h OGD, and 4 h reperfusion in the presence of IL-1β (OGD + R + IL-1). Immunocytochemical images for ZO-1 (**a**) were analysed with ImageJ to measure total intensity of ZO-1 fluorescence (**b**) and ratio of mean cytoplasmic fluorescence intensity to mean membranous ZO-1 fluorescence intensity (**c**). TEER measurements were carried out at the end of each treatment (**d**). Data are shown as mean ± standard deviation from three independent experiments carried out on separate cultures. Data were statistically analysed by one-way ANOVA and Bonferroni’s post hoc tests to compare all data sets. *p < 0.05, **p < 0.01, and ***p < 0.001 vs. normoxia. The *scale bar* in **a** represents 100 μm

In order to investigate the effect of hypoxia and/or inflammation on BBB integrity, we first tested the effect of OGD and/or IL-1 on endothelial cell morphology, as well as expression and cellular localisation of specific tight junction proteins involved in maintaining BBB integrity, namely occludin and ZO-1. OGD alone (2.5 h without reoxygenation) had no effect on endothelial cell morphology (demonstrated by a lack of change in cell circularity, Fig. 1a, b), had no effect on occludin expression (Fig. 1a, c) or cellular localisation (Fig. 1d), and had no effect on ZO-1 expression (Fig. 1a, c) or cellular localisation (Fig. 1d). In contrast, OGD (2.5 h) followed by 4 h reoxygenation induced a marked and significant change in endothelial cell morphology, with cells becoming rounder, compared to normoxic conditions (Figs. 2a and 3a). This effect was quantified by measuring the index of cell circularity and was found to be significant (Fig. 2b). Interestingly, IL-1β treatment alone (4 h) also induced a significant change in endothelial cell morphology similar to that seen after OGD and reoxygenation (Figs. 2a, b and 3a), whilst no change in cellular morphology was seen after OGD and reoxygenation in the presence of IL-1β (Figs. 2a, b and 3a).

OGD followed by 4 h reoxygenation in the absence or presence of IL-1β, or IL-1β treatment alone, had no effect on the total expression levels of occludin or ZO-1, compared to normoxia (Figs. 2c and 3b). In contrast, OGD and IL-1β induced differential relocalisation of tight junctions proteins, as assessed by the ratio of cytoplasmic/membranous immunostaining; for occludin, OGD and IL-1β treatment induced an increase in cytoplasmic/membranous ratio by 2.4-fold and 2.6-fold, respectively, whereas OGD and IL-1β combined induced a 6.3-fold increase in cytoplasmic/membranous ratio (Fig. 2d). For ZO-1, IL-1β induced a significant increase in the cytoplasmic/membranous ratio by 2.1-fold. OGD had no effect on the cytoplasmic/membranous ratio, whilst IL-1β and OGD combined induced a 1.7-fold increase in the cytoplasmic/membranous ratio (Fig. 3c).

We also investigated the effect of OGD and IL-1 on the TEER of the in vitro BBB model. OGD followed by 4 h reoxygenation had no significant effect on the TEER compared to normoxia (Fig. 3d). In contrast, IL-1β alone significantly reduced the TEER compared to control (normoxia), whilst the decrease in TEER observed after IL-1β treatment in the presence of OGD was also significant (Fig. 3d).

### OGD and IL-1 induce differential changes in endothelial expression of key ECM molecules in vitro

**Fig. 4:**
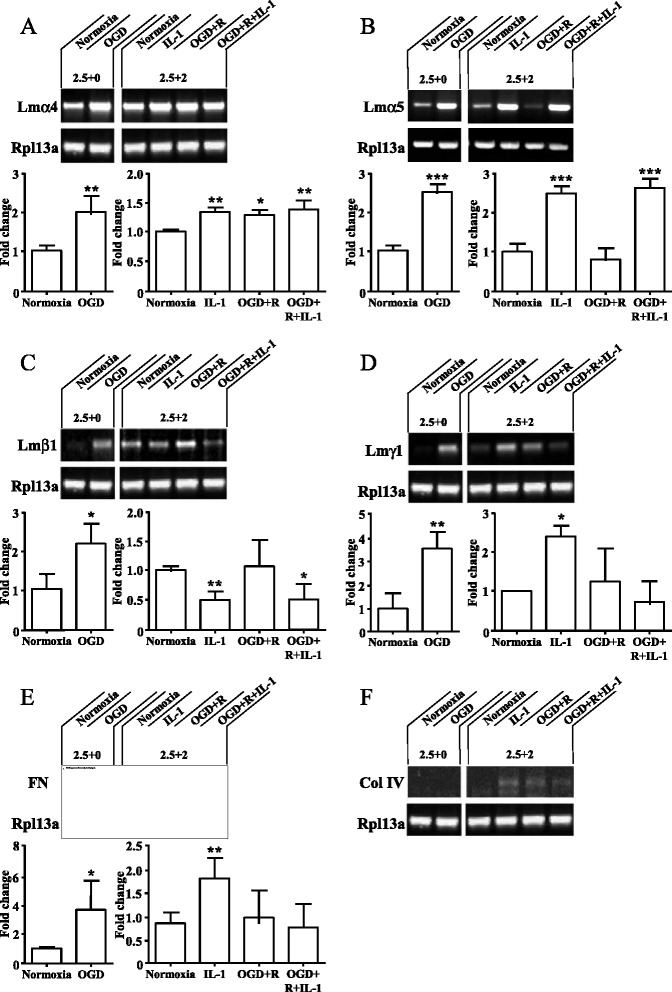
Primary rBEC cultures on Col I-coated Transwell® inserts were subjected to normoxia, or OGD (2.5 h) with (2.5 + 2, OGD + R) or without (2.5 + 0) reoxygenation for 2 h, and in the absence or the presence of IL-1β for 2 h. Total RNA was extracted, and RT-PCR was carried out for *lmα4* (**a**), *lmα5* (**b**), *lmβ1* (**c**), *lmγ1* (**d**), *fn* (**e**), *col4* (**f**), and the housekeeping gene *Rpl13a* (ribosomal protein L13A). Products were resolved on an ethidium bromide-containing 1.5 % (*w/v*) agarose gel by electrophoresis and then quantified using GE healthcare ImageQuant™. Data are shown as mean ± standard deviation from three independent experiments carried out on separate cultures. Data were statistically analysed by one-way ANOVA and Bonferroni’s post hoc tests to compare all data sets. *p < 0.05, **p < 0.01, and ***p < 0.001 vs. normoxia

**Fig. 5:**
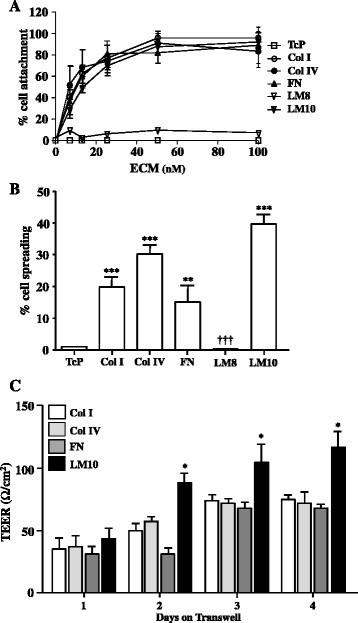
Primary rBECs were seeded onto tissue culture plastic pre-coated with BSA (TcP) or increasing concentrations of Col I, Col IV, FN, LM8, or LM10 for 90 min, and cell attachment was measured and expressed as percentage of cell number seeded (**a**). Primary rBECs were seeded onto wells coated with 100 nM BSA (TcP), Col I, Col IV, FN, LM8, or LM10 for 120 min, and cell spreading was measured and expressed as percentage of cell number seeded (**b**). Primary rBECs seeded onto Col I-, Col IV-, FN-, or LM10-coated (100 nM) Transwell® inserts were cultured for 1 to 4 days, after which TEER was measured (**c**). Data are shown as mean ± standard deviation from three independent experiments carried out on separate cultures. Data were statistically analysed by one-way ANOVA and Bonferroni’s post hoc tests to compare all data sets. For **b**, **p < 0.01 and ***p < 0.001 vs. TcP; ^†††^ *p* < 0.001 vs. Col I. For **c**, *p < 0.05 vs. Col I

Although FN, Col IV, LM8 (α4β1γ1) and LM10 (α5β1γ1) are key ECM molecules known to be associated with the BBB [13, 14], the specific effects of hypoxia and/or inflammation on expression of these ECM molecules by endothelial cells is unknown. We have used RT-PCR to assess the effect of OGD and/or IL-1β on gene expression of FN (*fn*), Col IV (*col4*), LM8 (*lmα4*, *lmβ1*, *lmγ1)* and LM10 (*lmα5*, *lmβ1*, *lmγ1*) in endothelial cells of our in vitro BBB model. OGD alone (2.5 h without reoxygenation) significantly increased expression of *lmα4*, *lmα5*, *lmβ1*, *lmγ1* and *fn* compared to normoxia (Fig. 4a–e). *Col4* was not detected in control normoxic cells, and OGD alone (2.5 h) had no effect on *col4* expression (Fig. 4f). The increased expression of *lmα5*, *lmβ1*, *lmγ1* and *fn* observed after OGD (2.5 h) was markedly reduced to normoxic levels following a period of 2 h reoxygenation, whilst *lmα4* expression remained unchanged (Fig. 4a–e). Although expression was very low, a slight increase in *col4* expression was observed in response to OGD and reoxygenation (Fig. 4f). Interestingly, IL-1β treatment alone (2 h) slightly induced expression of *col4* (Fig. 5f) but significantly increased expression of *fn*, *lmα4*, *lmα5* and *lmγ1*, whilst *lmβ1* expression was significantly reduced (Fig. 4a–e).

Finally, a combination of OGD with reoxygenation in the presence of IL-1β (2 h) significantly increased expression of *lmα4* and *lmα5* (Fig. 4a, b), and significantly reduced expression of *lmβ1* (Fig. 4c), compared to normoxia, although change in expression levels was similar to that of IL-1β treatment alone, demonstrating no synergic effect of OGD and IL-1β conditions. Expression of *fn*, *lmγ1*, and *col4* was unchanged after OGD and reoxygenation in the presence of IL-1β and was similar to expression seen in normoxia conditions (Fig. 4d–f).

### LM10 is a key regulator of BBB integrity

Since increased expression of ECM molecules was found to be associated with hypoxia and inflammation*,* we next hypothesised that such ECM protein expression could play a key role in re-establishing BBB integrity, which was lost by exposure to IL-1. In this set of experiments, we have focussed on IL-1 because of the marked and consistent effect of IL-1β on BBB dysfunction (Figs. 2 and 3).

We first investigated whether ECM molecules in vitro trigger endothelial cell adhesion. rBECs seeded onto Col I, Col IV, FN, or LM10 (ECM molecules chosen based on their increased expression induced by IL-1β and/or OGD*)*, showed increased cell adhesion in an ECM concentration-dependent manner compared to BSA-coated tissue culture plastic (Fig. 5a). Surprisingly, rBECs failed to adhere onto LM8. Similar effects were obtained on cell spreading, where significant cell spreading was observed when rBECs were seeded onto Col I, Col IV, FN, or LM10, but not on LM8 (Fig. 5b). Using a selective functional blocking antibody, we found that cell adhesion onto Col I, Col IV, and FN was integrin β1-dependent whereas cell adhesion onto LM10 was integrin β1-independent (data not shown).

**Fig. 6:**
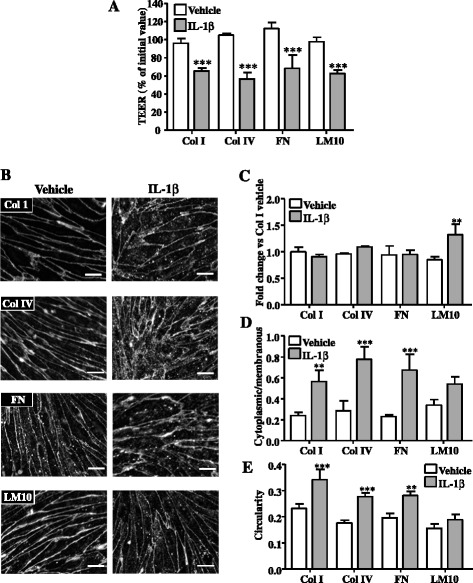
Primary rBECs seeded onto Col I-, Col IV-, FN-, or LM10-coated (100 nM) Transwell® inserts were cultured for 4 days, after which cultures were treated with vehicle or IL-1β for 4 h. TEER measurements were carried out at the end of each treatment (**a**). Immunocytochemical images for occludin (**b**) were analysed with ImageJ to measure total intensity of occludin fluorescence (**c**), ratio of mean cytoplasmic fluorescence intensity to mean membranous occludin fluorescence intensity (**d**), and cell circularity (**e**). Data are shown as mean ± standard deviation from three independent experiments carried out on separate cultures. Data were statistically analysed by one-way ANOVA and Bonferroni’s post-hoc tests to compare all data sets. *p < 0.05, **p < 0.01, and ***p < 0.001 vs. vehicle. The *scale bar* in **a** represents 10 μm

Interestingly, we found that LM10 specifically increased the TEER and could therefore contribute to BBB integrity; indeed, TEER of rBEC cultures increased from day 1 to day 4 of culture when cells were seeded onto Col I, Col IV, or FN, but there was no significant difference between the TEER of the rBEC monolayers grown on all these ECM proteins at any time point (Fig. 5c). In contrast, from day 2 to day 4 of culture in vitro, rBEC monolayers grown on LM10-coated Transwell® inserts had significantly higher TEER compared to the TEER of the monolayers grown on Col I-coated Transwell® inserts. These observations demonstrate that ECM molecules have differential effects on cell adhesion and TEER and identify LM10 as a key ECM molecule that could contribute to BBB repair after inflammatory injury. To test this hypothesis, we have assessed the effect of ECM proteins in the cellular response to IL-1-induced BBB dysfunction in vitro. Although none of the ECM molecules (including LM10) reversed the effect of IL-1β on TEER (Fig. 6a), we found that LM10 specifically reversed key hallmarks of BBB dysfunction induced by IL-1β in our BBB in vitro model; IL-1β had no effect on occludin expression when cells were plated onto Col I, Col IV, or FN (Fig. 6b, c). In contrast, occludin expression was significantly increased by IL-1β when cells were plated onto LM10 (Fig. 6b, c). Furthermore, significant changes in occludin cellular localisation (shifting from the membranous to the cytoplasmic compartment) were induced by IL-1β in cells plated on Col I, Col IV, or FN; however, no significant change in cellular localisation of occludin was observed in cells plated on LM10 (Fig. 6b, d). Furthermore, IL-1β treatment significantly affected cellular morphology, causing an increase in the circularity index of cells plated on Col I, Col IV, and FN, with no such change in morphology observed in cells plated on LM10 (Fig. 6b, e).

## Discussion

The BBB undergoes dynamic physical and functional changes after cerebral ischaemia, including degradation of key ECM molecules of the basement membrane, and loss of physical interactions between the basement membrane, endothelial cells, and perivascular astrocytic endfeet. These events lead to increased BBB permeability and are the primary events allowing leucocyte infiltration which is known to contribute to damage (see [15] for review). These events occur alongside the development of a potent vascular inflammatory response primarily initiated by IL-1 acting on endothelial cells [10]. Although our previous study has identified a dynamic process of vascular ECM remodelling after ischaemic injury [8], the precise mechanisms and functional consequences of those changes have largely remained unexplored. Using an in vitro model of the BBB, we showed here for the first time that hypoxia and IL-1 are key triggers for BBB dysfunction, that this process is accompanied by increased expression of key brain vascular ECM molecules, and that LM10 is a key ECM molecule implicated in BBB repair after inflammatory injury.

We first demonstrated that, although OGD and IL-1 treatment caused no significant changes in expression levels of occludin and ZO-1, both OGD reoxygenation and IL-1 induced marked changes in their cellular localisation, with translocation from the plasma membrane to the cytosolic compartment, resulting in decreased levels at the tight junction. This was accompanied with a marked change in cellular morphology as well as significant decreases in BBB permeability. The effect of hypoxia [16, 17] and IL-1 [18] on increasing BBB permeability in vitro has been previously reported and is consistent with BBB dysfunction seen in ischaemic and inflammatory conditions such as stroke [19]. However, our study is the first to show that cellular relocalisation of key tight junction proteins could be the primary mechanism of BBB disruption. Furthermore, the change in cellular morphology indicated cell swelling, which is in agreement with changes seen in vivo where vasogenic oedema characterised by endothelial cell swelling occurs after cerebral ischaemia [17]. In our study, we have used occludin and ZO-1 expression/cellular localisation as established markers of BBB integrity. However, one cannot exclude the possibility that other tight junction molecules are also affected. Amongst those, claudin-5 is another key tight junction molecule, and two very recent studies using similar in vitro approaches to ours found decreased claudin-5 expression concomitant with decreased occludin expression after OGD and reoxygenation [20, 21].

We further showed that the expression of key ECM molecules known to be associated with the cerebrovasculature, namely FN (*fn*), Col IV (*col4*), LM8 (*lmα4*, *lmβ1*, *lmγ1*), and LM10 (*lmα5*, *lmβ1*, *lmγ1*), was strongly increased in endothelial cells exposed to OGD; increased expression was transient since expression of all ECM molecules tested returned to basal level after 2 h of reoxygenation. These results are in agreement with previous studies showing that expression of FN, Col IV, and LM isoforms is increased after hypoxic conditions in vitro in endothelial cells [3] and in vivo [8, 22]. However, our findings further identified LM8 and LM10 to be markedly induced by hypoxia, which has not been reported previously. Importantly, we found that IL-1 is a key inducer of endothelial cell ECM expression in vitro, since expression of *col4*, *fn*, *lmα4*, *lmα45*, and *lmyγ1* was strongly increased by IL-1 treatment, whilst *lmβ1* expression was slightly reduced. The contribution of IL-1 to endothelial cell activation has been extensively reported previously [10, 23], but our study is the first to demonstrate that IL-1 also upregulates endothelial cell expression of ECM molecules, and suggests that both ischaemia and subsequent inflammation can promote ECM expression in the cerebrovasculature after stroke. This is in agreement with other studies showing increased ECM expression in the brain vasculature after stroke [3, 24], which has been proposed as a key step for ECM remodelling and BBB recovery.

In an attempt to understand the pathophysiological consequences of increased expression of ECM molecules after hypoxia and inflammation in endothelial cells, we have tested the effect of the same recombinant ECM proteins on endothelial cell adhesion and markers of BBB dysfunction induced by IL-1. We found strong adhesion of cerebrovascular endothelial cells to Col I, Col IV, FN, and LM10, but not to LM8. Interestingly, we found that the TEER of the endothelial cell monolayer strongly increased over the time of culture when cells where plated onto Col I, Col IV, or FN, and that this increase was further potentiated when cells were plated onto LM10, suggesting that this ECM molecule has a very important role for the establishment of BBB integrity. In support of this, we also found that LM10 specifically reversed key features of BBB dysfunction in vitro, including occludin cellular relocalisation and cell swelling. The role of LM10 in peripheral immune cell transmigration has been demonstrated previously [13], and our study is the first to demonstrate that LM10 could play an important role in BBB recovery and repair. The effect of LM10 on IL-1-induced BBB dysfunction was, however, only partial since LM10 failed to affect IL-1-induced decreased TEER in vitro. The reason for this is unclear, but one likely possibility is that whilst LM10 may reverse key microstructural changes at the tight junction, some possibly undetected nanostructural changes that could affect the TEER might not be initiated by plating the cells on LM10 only. This may indicate that although LM10 appears to trigger partial recovery of BBB dysfunction induced by IL-1 in vitro, other key ECM molecules are probably required to promote full BBB recovery after injury in vivo. Taken together, our results suggest that ECM remodelling, through over-expression of key ECM molecules at the cerebrovasculature, may be a critical step for the restoration of BBB integrity after injury. Recent in vivo studies found that increased expression of ECM molecules (including LM isoforms) correlates with restoration of BBB functions [25–27], and the in vivo relevance of our finding, particularly in relation with the contribution of LM10 to BBB repair, remains to be determined.

## Conclusions

Our data suggest a possible new mechanism by which OGD and IL-1 induce BBB dysfunction as well as changes in cerebrovascular ECM protein expression, amongst which LM10 appears to play a key role in partial BBB repair. Our study therefore identifies this ECM molecule as a major target for brain tissue repair after stroke and other cerebrovascular inflammatory disorders. This study was performed entirely in vitro and was designed as a proof of concept study to show the potential role of LM10 in BBB repair. However, further studies are necessary to determine the relevance of LM10 function in brain injury in vivo and the possible therapeutic relevance of targeting LM10 for functional recovery.

## Abbreviations

BBB: blood–brain barrier
BEC: brain endothelial cell
Col: collagen
DIV: days in vitro
ECM: extracellular matrix
FN: fibronectin
IL-1: interleukin-1
LM: laminin
OGD: oxygen-glucose deprivation
rBEC: rat brain endothelial cell
ZO-1: zona occludens-1
